# Epiploic gonadal vein as a new bypass route for extrahepatic portal venous obstruction: report of a case

**DOI:** 10.1186/s40792-015-0112-7

**Published:** 2015-10-23

**Authors:** Tatsuaki Sumiyoshi, Yasuo Shima, Takehiro Okabayashi, Yuji Negoro, Akihito Kozuki, Jun Iwata, Yuichi Saisaka, Teppei Tokumaru, Toshio Nakamura, Sojiro Morita

**Affiliations:** Department of Gastroenterological Surgery, Kochi Health Sciences Center, 2125 Ike, Kochi City, Kochi 781-8555 Japan; Department of Gastroenterology, Kochi Health Sciences Center, Kochi, Japan; Department of Diagnostic Pathology, Kochi Health Sciences Center, Kochi, Japan; Department of Radiology, Kochi Health Sciences Center, Kochi, Japan

**Keywords:** Extrahepatic portal venous obstruction, Shunt surgery, Epiploic vein, Gonadal vein

## Abstract

A 61-year-old man was referred to our hospital to treat extrahepatic portal venous obstruction. Endoscopic injection sclerotherapy (EIS) was performed for the esophageal varices; however, the patient returned with massive hematemesis from gastric varices 6 months after treatment. Although the varices were treated with EIS, gastric devascularization and splenectomy concomitant with shunt surgery were required to treat uncontrollable, frequent diarrhea and abdominal distension. Because the splenic vein, left gastric vein, left portal vein, and inferior vena cava were inadequate for anastomosis, an epiploic gonadal vein bypass was performed. The bypass graft remains patent 7 months after surgery, and the patient is in good health without any clinical symptoms. We describe a new bypass route for extrahepatic portal venous obstruction.

## Background

Extrahepatic portal venous obstruction (EHPVO) is a disorder characterized by chronic blockage of portal venous flow that results in portal hypertension, and its associated elevated portal venous pressure leads to gastroesophageal variceal hemorrhage [1–4]. Shunt surgery is indicated for refractory or complicated cases of this disease [5–13]. Several surgical shunts have been reported, and currently, mesenteric-left portal vein bypass (MLPVB) is the primary shunt used for EHPVO because it is the most physiologic shunt and it restores hepatic blood flow [5–10, 12]. However, MLPVB is limited to those with favorable anatomy, and it cannot be used in cases in which the left portal vein (LPV) is occluded or strictured. We report a case of a severe adult-onset case of EHPVO in which epiploic gonadal vein bypass was effective.

## Case presentation

### Patient

A 61-year-old male.

#### Chief complaint

Severe diarrhea and abdominal distention.

#### Past history

Bilateral common iliac artery stenting for the arteriosclerosis obliterans at the age of 57.

#### Present illness

**Fig. 1:**
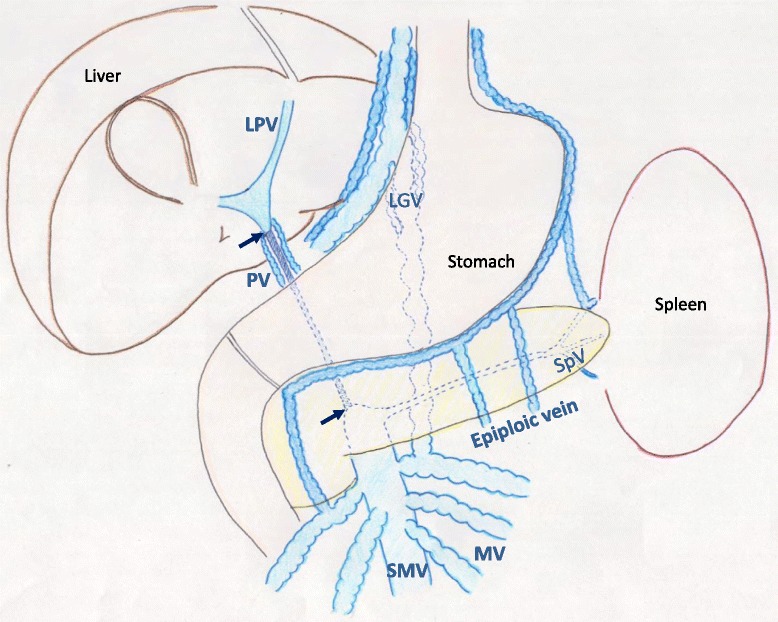
Schema of extrahepatic portal venous obstruction. Extrahepatic main portal vein (PV) was occluded (*between black arrows*). The left portal vein was constricted, although it was perfused by cavernous transformation, and the patient’s splenic vein (SpV) was also constricted. The left gastric vein (LVG) and mesenteric vein (MV) were markedly dilated. *SMV* superior mesenteric vein

A 61-year-old male presented with severe diarrhea and abdominal distention. Computed tomography (CT) showed EHPVO, and he was referred to our hospital for treatment. Although laboratory test results showed a low albumin level of 2.7 g/dL and a decreased platelet count of 9.8 × 10^4^/μL due to splenomegaly, liver function tests, including the serum ammonia level, were normal. The protein C level (65 %) and protein S level (105 %) were normal. Further, the patient had no previous history of abdominal surgery or trauma, and the cause of EHPVO was suspected to be idiopathic. Upper gastrointestinal endoscopy revealed linear varices without a red color sign from gastric cardia to the middle part of the esophagus, and endoscopic injection sclerotherapy (EIS) was performed. He presented 6 months after the endoscopic treatment with massive hematemesis and was readmitted to our hospital. Endoscopy showed bead-shaped moderate varices with a red color sign and active bleeding from gastric varices. EIS was successfully performed. CT showed an occluded extrahepatic portal vein and markedly dilated left gastric vein (LGV) and mesenteric veins (Fig. 1). Cavernous transformation around the main portal vein and inferior vena cava (IVC) was seen. Because the patient’s activity of daily life was severely impaired by frequent diarrhea and abdominal distention and because the risk of rebleeding was high, gastric devascularization and splenectomy concomitant with shunt surgery was planned. Before the surgery, the markedly dilated LGV and IVC were the candidates for the proximal and distal sites of the shunt, respectively, because the splenic vein (SpV) and LPV had become constricted and were inadequate for anastomosis (Fig. 1).

#### Surgical findings

**Fig. 2:**
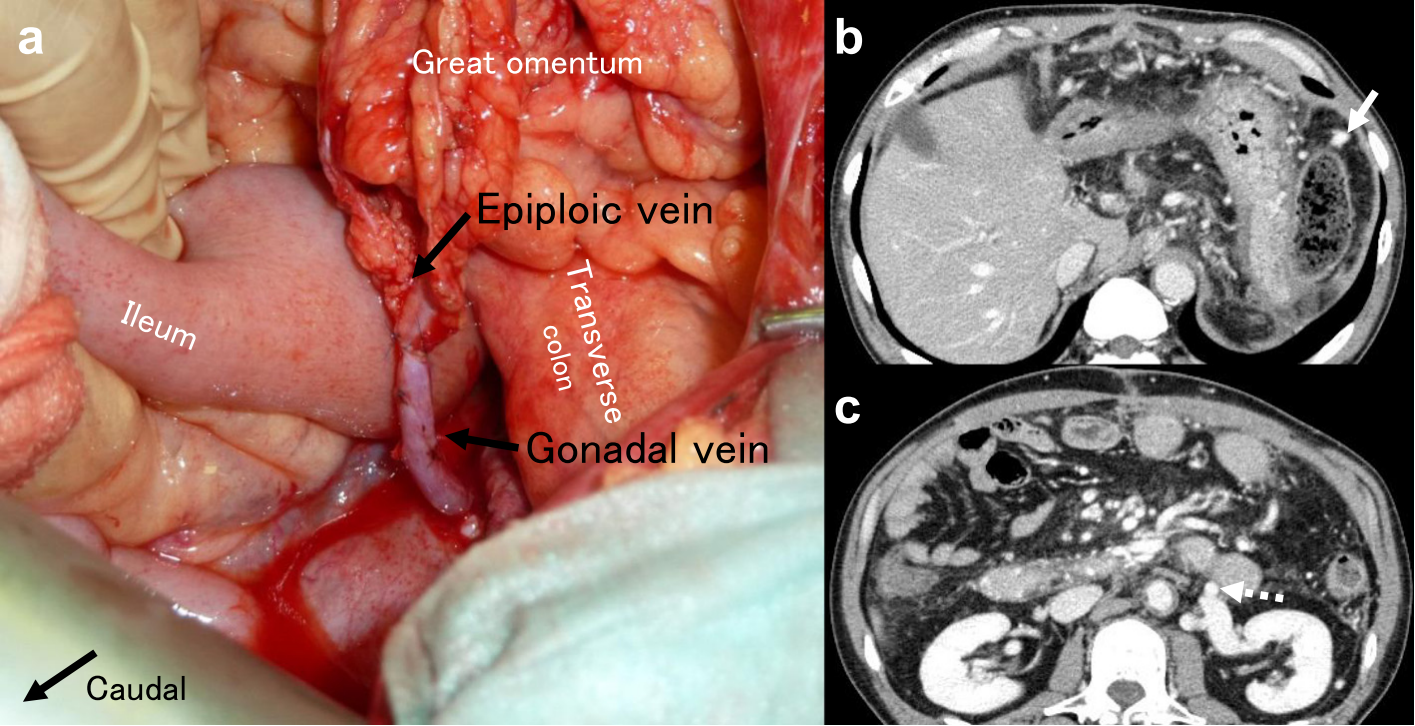
Epiploic gonadal bypass. **a** Intraoperative photograph showing end-to-end anastomosis of the epiploic and left gonadal veins. **b**, **c** Postoperative computed tomography showing patent and well-dilated epiploic vein (*solid arrow*) and left gonadal vein (*dotted arrow*)

Firstly, gastric devascularization and splenectomy were performed. However, the dilatation of epiploic vein in the greater omentum got worse after the devascularization, and shunt surgery was planned. Nets of collateral vessels were found circling the LGV and IVC, and it was difficult to handle these vessels. Severe inflammation after EIS was also seen around the LGV. The epiploic vein was markedly dilated, so it was chosen as the proximal site of the bypass. Instead of using the IVC for the distal site of the bypass, the left gonadal vein was chosen. The anastomosis was constructed end-to-end with a single running suture using 6–0 Prolene (Fig. 2).

#### Postoperative course

**Fig. 3:**
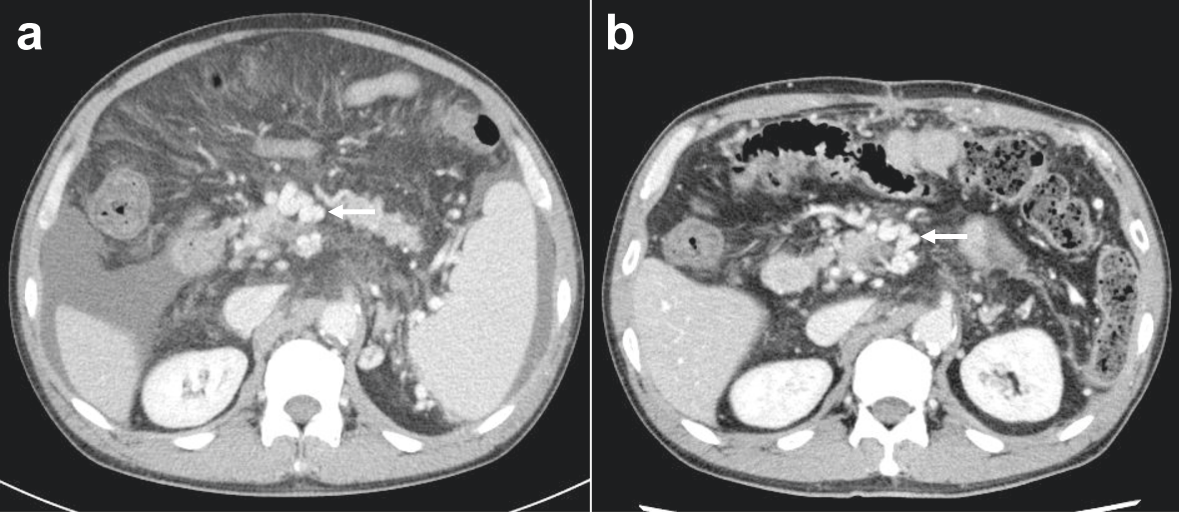
Preoperative CT (**a**) and postoperative CT (**b**). CT 3 months after the surgery showed disappearance of ascites and mesenteric edema. The dilatation of the mesenteric vein was reduced (*white arrow*)

The postoperative course was uneventful, and the patient was discharged 14 days after surgery. CT 3 months after the surgery showed disappearance of ascites and mesenteric edema (Fig. 3). The dilatation of the mesenteric vein was also reduced. The bypass graft remains patent 7 months after surgery, and the patient is in good health without any clinical symptoms.

### Discussion

EHPVO causes portal hypertension, and up to 70 % of the etiological factors of this disease remain idiopathic [1–3]. Almost 90 % of children with EHPOV present with variceal bleeding, and the remaining 10 % present with splenomegaly [2]. Unlike patients with chronic liver disease, those with EHPVO have preserved liver function. Therefore, mortality is mainly due to variceal bleeding, and endoscopic therapy, either EIS or endoscopic variceal ligation, is indicated to treat the esophageal varices [2]. Endoscopic therapy has been reported to be effective for controlling acute bleeding from esophageal varices in 80–90 % of patients [1]. However, endoscopic therapy does not decrease the elevated portal venous pressure, or, as in the current case, resolve other symptoms due to portal venous hypertension, such as ascites and diarrhea. Therefore, in refractory or complicated cases, shunt surgery is indicated. High long-term patency rates, ranging from 89 to 97 %, have been reported [6]. To prevent the shunt thrombosis, low doses of heparin during the first week followed by antiplatelet agents for 3–6 months is recommended [6]. A successful shunt decreases the portal venous pressure and improves most of the clinical symptoms. Shunt surgery is classified as “selective” or “nonselective”. Selective shunts are usually recommended because nonselective shunts, such as the mesocaval shunt, have been associated with a significant risk of hepatic encephalopathy compared with selective shunts [6]. Distal splenorenal shunt and MLPVB have been reported to be the representative selective shunts [12, 13]. The distal splenorenal shunt, first described in 1967, has a lower incidence of clinically significant post-shunt encephalopathy [13]. One problem with the splenorenal shunt is that it cannot be performed in almost one-third of cases due to a small or blocked SpV [14, 15]. Another selective shunt, MLPVB, was first performed by de Ville de Goyet et al. in 1992 in a 3-year-old patient with extrahepatic portal vein thrombosis after a partial liver transplant [12]. This shunt restores mesenteric blood flow to the liver through the Rex recess, and it is the most physiologic shunt. In the current case, however, preoperative CT showed the SpV and LPV were narrowed. Further, during laparotomy, an enlarged LGV, which was the first candidate for the proximal site of anastomosis, could not be handled and mobilized because of severe inflammation and nets of collateral vessels around it. Therefore, the epiploic and gonadal veins were chosen as anastomosis sites in this case. These vessels were much easier to handle and mobilize than the LGV and IVC. Autogenous epiploic and gonadal veins have not been used in shunt procedures to date, and this is the first case report. Although MLPVB and distal splenorenal shunts remain the first candidates for shunt surgery, epiploic gonadal vein bypass might be an option, especially in cases with narrowed LPV and SpV.

## Conclusions

Epiploic gonadal vein bypass could be a new candidate of bypass route for EHPVO patients.

## Consent

Informed consent was obtained from the patient for publication of this Case Report and any accompanying images.

## Abbreviations

CT: computed tomography
EHPVO: extrahepatic portal venous obstruction
EIS: endoscopic injection sclerotherapy
IVC: inferior vena cava
LGV: left gastric vein
LPV: left portal vein
MLPVB: mesenteric-left portal vein bypass
SV: splenic vein
